# A case study of using the He Pikinga Waiora Implementation Framework: challenges and successes in implementing a twelve-week lifestyle intervention to reduce weight in Māori men at risk of diabetes, cardiovascular disease and obesity

**DOI:** 10.1186/s12939-020-01222-3

**Published:** 2020-06-22

**Authors:** John Oetzel, Moana Rarere, Ray Wihapi, Carey Manuel, Jade Tapsell

**Affiliations:** 1 Waikato Management School, University of Waikato, Hamilton, New Zealand; 2 National Institute of Demographic and Economic Analysis (NIDEA), University of Waikato, Hamilton, New Zealand; 3 Poutiri Trust, Te Puke, New Zealand

**Keywords:** Lifestyle intervention, Implementation framework, Diabetes prevention, Indigenous communities, Māori

## Abstract

**Background:**

Māori men have stark health inequities around non-communicable diseases. This study describes the case of a partnership attempting to develop and implement a culturally centred intervention through a collaborative partnership to potentially address the inequities. In particular, the partnership followed a participatory, co-design approach using the He Pikinga Waiora (HPW) Implementation Framework; the study presents lessons learnt in addressing health inequities following this framework.

**Methods:**

The partnership involved a university research team and a Māori community health provider. They engaged with other stakeholders and several cohorts of Māori men through a co-design process to adapt a 12-week lifestyle intervention. The co-design process was documented through meeting notes and interviews with partners. Two cohorts participated in separate single group pre-intervention/post-intervention designs with multi-method data collection. Key outcome measures included weight loss, self-reported health, physical activity, and nutrition. Post-intervention data collection included qualitative data.

**Results:**

The co-design process resulted in a strong and engaged partnership between the university team and the provider. There were significant challenges in implementing the intervention including having two additional partner organisations dropping out of the partnership just after the initial implementation phase. However, a flexible and adaptable partnership resulted in developing two distinct lifestyle interventions run with 32 Māori men (in two different cohorts of 8 and 24). All but one in the first cohort completed the programme. The first cohort had a modest although statistically insignificant improvement in weight loss (d = 1.04) and body mass index (BMI; d = 1.08). The second cohort had a significant reduction in weight loss (d = 1.16) and BMI (d = 1.15). They also had a significant increase in health-related quality of life (d = 1.7) and self-rated health (d = 2.0).

**Conclusion:**

The HPW Framework appears to be well suited to advance implementation science for Indigenous communities in general and Māori in particular. The framework has promise as a policy and planning tool to evaluate and design interventions for chronic disease prevention in Indigenous communities. Despite this promise, there are structural challenges in developing and implementing interventions to address health inequities.

**Trial registration:**

Retrospectively registered, Australia New Zealand Clinical Trials Registry, ACTRN12619001783112.

## Introduction

Health inequities in chronic, non-communicable diseases between Māori and non-Māori in Aotearoa (New Zealand) are persistent and compelling [1, 2], and consistent with inequities faced by Indigenous people in other countries [3]. For example, 7.2% of Māori have diabetes compared to 5.1% of Pākehā (New Zealand European) [4]. Further, Māori have 1.8 times greater health burden than non- Māori and a 9 year lower average life expectancy [2]. Racism along with unjust distribution of social determinants of health are root causes of these inequities [5]. Additionally, the lack of commitment in the past by the New Zealand Government towards obligations under Te Tiriti o Waitangi (The Treaty of Waitangi; the founding document for New Zealand that outlined the relationships between Māori and non- Māori colonisers) is a fundamental driver of the unequal distribution of the determinants of health and inaction in the face of need [6].

Māori men have similar rates of inequities for diabetes and cardiovascular diseases as the larger population statistics [7] that result, in part from challenges to engaging in lifestyle changes and lifestyle interventions due to commitments to family and other priorities along with social determinants and environmental factors [8, 9]. There is ample evidence from systematic reviews that lifestyle interventions involving physical activity and nutrition components are effective at preventing diabetes and cardiovascular disease along with reducing weight [10–15]. However, evidence-based interventions may not be effective in Indigenous communities without adapting the intervention to fit the target community; for example, a lifestyle intervention for Māori failed to recruit and sustain participants [16].

An oft-used approach for adapting evidence-based lifestyle interventions is a participatory approach such as community-based participatory research (CBPR) [17]. CBPR is an approach that equitably involves community and academic researchers in all phases of the research [18]. Using CBPR methods, there are numerous studies that demonstrate the effectiveness of culturally adapted and/or created lifestyle interventions on risk factors for diabetes, cardiovascular disease, and obesity for Indigenous communities in general [19–24] and Māori communities in particular [25–27].

The current study used a particular CBPR-based approach that was developed for Māori. The He Pikinga Waiora (Enhancing Wellbeing, HPW) Implementation Framework has Indigenous self-determination and knowledge at its core and consists of four elements: cultural-centeredness, community engagement, systems thinking, and integrated knowledge translation [17, 28]. All elements have conceptual fit with Kaupapa Māori aspirations (i.e., Indigenous knowledge creation, theorizing, and methodology) and all elements have been shown to demonstrate positive implementation outcomes [28]. In brief, it is a participatory approach that equitably involves community and academic partners to co-design, co-implement, and co-evaluate health interventions. It ensures that cultural and community perspectives are integrated in interventions by having community members define problems and solutions. It involves shared decision making and authority of the project between communities and academics. Further, it engages with end users (e.g., policy makers and practitioners who will use/support interventions) to help support the sustainability of the intervention. Finally, it takes a systems perspective to locate interventions into a complex web of people, practices, and organisations.

The purpose of this study is to present a case about a partnership involving a Māori community health provider to develop a 12-week lifestyle intervention using the HPW co-design/participatory research process. We hoped to demonstrate the usefulness of the HPW framework for developing a culturally-adapted intervention to fit the needs of the participants and communities. There were two research aims:

- Aim 1: To document and present implementation challenges and successes that resulted during the co-design/participatory process.
- Aim 2: To present the outcomes from two different lifestyle interventions that were created during the co-design process.

The second aim included two different interventions as the partnership explored several options to target Māori men in their communities identified during the co-design process.

## Methods

### Research partners

A university research team partnered with Poutiri Trust (Poutiri) for this project. Poutiri is a Māori Development Organisation that provides a range of health and social services to a predominantly Māori client base. These two organisations also worked with additional stakeholders during the co-design process.

### Interventions

There were two cohorts with slightly different approaches that resulted from the co-design process; both targeted Māori men at risk for Type 2 diabetes and related conditions including cardiovascular disease and obesity. Both interventions used components from various lifestyle interventions (physical activity and nutrition) primarily the Diabetes Prevention Program [29, 30]. Other elements of the intervention were developed through the co-design process although one key element (use of a peer or community health worker for support) has grounding in the extant literature [31, 32]. These elements identified during the co-design process came from the participants as being motivating factors for their participation; differences between the two cohorts reflect this process.

The first cohort was organised around a Christian church although participation was open to non-members through the networks of the members and through a Facebook call. These included both men and women with the belief that including women would help link their partners to the intervention. The minimal criteria to enrol in the first cohort through the church and Facebook call were to be an adult Māori male with a Body Mass Index (BMI) of 25 or higher or an adult member of the whānau (extended family) of a participant.

The second cohort was organised through a trainer at a local gym. He recruited community members who met the eligibility criteria from his network and the networks of participants; most of the men were not active gym participants at the beginning of the programme. All participants were men in this cohort. For the second cohort, the eligibility criteria were restricted to Māori male with a Body Mass Index (BMI) of 25 or higher. The rationale for the recruiting locations was that the provider wanted to target groups of people they had not worked with previously; these groups were identified by stakeholders in the co-design process. Table 1 summarises the features of the interventions.

**Table 1:** Intervention Features

Element	Cohort 1	Cohort 2
Physical Activity	Four self-selected activity groups: a) walking group + box fit (moderate intensity) or Zuu fit (high intensity interval training) classes; b) walking group only; c) Box and/or Zuu fit classes; and d) self-organising group involving various activities including walking and touch rugby	Individually tailored consultations about physical activity (education, workout plans, and physical activity sessions); Delivered through whatever means was desired (e.g., phone, face-to-face, home visit)
Nutrition	Weekly one-hour didactic session	Weekly 30-min education delivered via a booklet
Who Delivered	Tuakana (senior mentor) who was also a participant	Kaiarahi (guide or community health worker)
Frequency	Three times per week for 1 h each session	Determined by the participant
Other Elements	Monthly prizes for greatest percentages of weight loss; Facebook group; Participant information booklets	Health screen with nurse (e.g., lipids, blood pressure, CVD risk) at baseline with referral to GPs if necessary

### Research design

The research design for each cohort (*n* = 8 and 24) was a single group pre-intervention/post-intervention design. A comparison group was originally planned, but was not feasible after one of the stakeholder organisations had to withdraw due to a funding crisis. The two cohorts were not directly compared because they were designed through a co-design process and thus had different features; sample size also limited direct comparison. The study was granted ethical approval through the University of Waikato Ethics Committee (15/202).

### Data collection

A multi-method approach was adopted for understanding the process of development and the outcomes from the intervention. The co-design process was documented through meeting notes and interviews with three of the research partners and two additional stakeholders. Thus, researcher insights are a key component of the methods. The outcomes included a variety of baseline and post-intervention measures conducted through self-report questionnaires, height and weight measurement and open-ended questions about the intervention experience (post-intervention). The relevant outcomes were identified during the co-design process through discussion among the research partners. Table 2 lists these measures. Three measures were not used in the post-intervention for the 2nd cohort to try to reduce response burden due to complaints from the men at the baseline survey. Other items not collected at follow-up were included to describe the sample.

**Table 2:** Outcomes Measures

Measure	Baseline	Post-Intervention
Height & Weight (BMI)	X	X
Self-reported health (1 item) [33]	X	X
Health related quality of life (HRQOL, 7 items) [34, 35]	X	X
Health service utilisation (6 items) [36]	X	X
Total days with 30 min moderate/15 min vigorous activity (1 item) [36]	X	X
Nutritional intake (9 items) [37]	X	X
Social support (2 items) [38]	X	X (cohort 1 only)
Readiness to change (3 items) [39]	X	X (cohort 1 only)
Self-efficacy to change (3 items) [39]	X	X (cohort 1 only)
New Zealand deprivation index (8 items) [40]	X	
Trust in institutions (7 items) [41]	X	
Cultural identity (2 items) [42]	X	
Demographics	X	
Open-ended questions: impact on health and that of their whānau, changes made and what they liked about the programme		X

### Data analysis

Demographic details were analysed using frequencies or mean/standard deviations. Constructs with multiple items were checked for internal consistency (Cronbach’s alpha) and with sufficient alpha were averaged for a scale score. All items used the original scale scoring except for self-rated health and HRQOL which were converted to 100-point scoring following the RAND method [43]. Data analysis for the outcome measures utilised paired sample t-tests with SPSS 25.0; effect size was also calculated [44]. Qualitative data were analysed with framework analysis to identify responses related to each area of interest by the team. Frequencies in each category were calculated [45].

## Results

### Co-design process

Table 3 summarises the co-design process and key events. In brief, the co-design process followed four stages. The first stage was initiating the relationship to define the project and identify the need for additional research/information. After this information was collected, the second stage involved exploratory co-design of the intervention with a range of stakeholders that resulted in identifying the target audience and target condition. The third stage involved a focused co-design process with interested stakeholders and the participants of the intervention. The final stage was the implementation of the two interventions.

**Table 3:** Co-design Process

Event	Date	Description
Initial scoping of project	1–6/2016	Held several meetings to build a relationship between the university team and Poutiri. We also identified shared goals and what work had been done in the community previously and needs for further information. Poutiri identified the importance of sustainability for any new intervention.
Developed HPW Implementation Framework [28]	2–9/2016	Reviewed the international literature and shared framework with stakeholders for feedback.
Created causal loop model	6–12/2016	Created a causal loop model of factors for prediabetes and diabetes following soft systems logic using stakeholder interviews; Prediabetes was the primary disease of interest initially.
Conducted patient interviews	6–12/2016	Poutiri conducted interviews of their own patients with pre-diabetes and diabetes to better understand facilitators and barriers to care [46].
Meetings with key Poutiri stakeholders	3–6/2017	Met with Poutiri Board of Trustees, Poutiri’s network or providers and the District Health Board to share findings from the previous year’s work and to further scope intervention and identify additional stakeholders.
Initial co-design meetings	7–8/2017	Held several co-design meetings following a design thinking framework to determine target audience, craft potential interventions and identify partners. Information from systems map and patient interviews was shared and integrated into interventions ideas. Stakeholders including representatives from two primary health organisations (PHO), a public health organisation, and Poutiri’s network of providers.
Advanced co-design meetings	8/2017–3/2018	Determined the key target population for these stakeholders should be Māori men because there were no existing contracts that targeted men; focus on pre-diabetes and related conditions particularly related to weight. We honed in on gang members and their whānau because they represented a group underutilising health services and were a group several stakeholder organisations wanted to reach. We held co-design meetings with men and their whānau led by Poutiri. Other key stakeholders were a social service organisation for gang members, a PHO, and a provider in Poutiri’s network.
Launch intervention	5/2018	Held a health fair and began recruitment for a lifestyle intervention with integrated care (i.e., nurse to triage health issues and refer to needed services; community health worker to provide lifestyle intervention and be a navigator; social services; activities for community health improvement all through a single place of contact). Unfortunately, it never gained traction. The PHO had originally committed 1 day a week of nurse’s time along with co-delivering the lifestyle intervention and collecting data from a different community as a comparison group. Unfortunately, they had to withdraw their full support, and re-commit their resources to more pressing priorities due to losing a significant proportion of their primary health practices and patients to another PHO. Additionally, the social service provider had internal governance challenges that required immediate attention, which meant they could no longer support the intervention in terms of co-delivery and access to the target population.
Re-design intervention for 1st cohort	8–9/2018	With an emphasis on research team flexibility to address changing conditions, we focussed on re-designing the intervention with Poutiri as the only community stakeholder. Community members had some input into a lifestyle intervention for men and their whānau. The focus became solely on the lifestyle intervention rather than integrated care due to time constraints. The cohort focused on physical activity with some nutritional information.
Implement intervention	9–12/2018	Intervention was implemented with the first cohort.
Re-design intervention for 2nd cohort	1–3/2019	The first cohort had a limited number of men so we redesigned the intervention with direct input from the target audience. The result was an individually-tailored intervention to allow for flexibility for full-time working men.
Implement intervention	3–6/2019	Intervention was implemented with the second cohort.

The key elements of the HPW framework were integrated to support the design and development of the implementation programme. We completed a process evaluation that demonstrated consistency with the HPW principles and that were used for reflection to improve the process [47]. The evaluation suggested that there were strengths in the participatory process (i.e., community engagement and culture-centredness), with some improvements in end user engagement (i.e., integrated knowledge translation) and systems thinking to support the sustainability of the intervention. One of the academic researchers was asked about whether the partnership followed the HPW principles:

*I think we did – well, certainly in committee engagement and cultured centredness we did really well. At the beginning, I think we did four out of five principles really well. I think it’s always been Kaupapa Maori focused. As I said, I think we could have done better in terms of IKT [integrated knowledge translation]. I think we did pretty well systems thinking, but those are the areas that we could have improved. I think we were spot on in terms of community engagement and culture centredness.*

One of the community researchers agreed with this sentiment in expressing what she thought worked well:

*Yeah, the model of co-design. Having that framework also sets really strong measures. It’s a proper framework that you can actually measure against [*i.e.*, clear standards for evaluating the quality of co-design]. You don’t have to do a lot of extra mahi (work) to figure out whether what you’re doing aligns with it.*

A second community researcher also supported these thoughts about following the framework and also reflected on the initial intervention:

*What we did initially with the group down there was amazing. If that would have got off the ground it would have got huge traction and that was no fault of anyone; it was again the environment at the time. But I think that approach and how you guys [academic researchers] approached it in accordance with your framework, perfect. Those other factors that made it tip over were out of our control.*

Reflecting on their own challenges as an organisation and working with other community organisations, the two community researchers identified some strengths of the team and potential improvements:

*The flexibility [is a strength]. I think organisationally, we needed to be more flexible … The bureaucracy. Yeah, like [other community organisations], we all needed to be a little more flexi. I think you guys were really flexi and made it work.*

The first community researcher identified challenges and potential learnings:

*I don’t think that anything that happened could have been planned for at the start; they were learnings that we had to have. But in terms of what it could have been, we were just talking about how we could have maintained better lines of communication, because as a community partner, we went through changes internally in the role. As a community researcher, I stepped right back out of the role once the new person went in and that meant that there was a loss of that knowledge and oversight … .so, our internal communication could have been better.*

This researcher also noted that the intervention itself resulted in learnings:

*In terms of the actual intervention, heaps of learnings, heaps of learnings for us. We went big knowing that we’ll either go big or go home and we’ve had to go home a little bit, but that hasn’t dissuaded us from doing it*.

In fact, the organisation decided to continue funding the 2nd intervention because of the positive results, the sense of ownership of the intervention and the responsibility to deliver a preventive programme for their community. This decision is consistent with their initial commitment to a sustainable intervention.

### Cohort 1

#### Demographics

A total of 43 participants enrolled for the first cohort and 79% were Māori with only 35% male. However, there were only 8 Māori men and results are presented only for them. The average age was just above 37 and the sample had relatively low food insecurity and deprivation scores. They expressed a middle attitude in regards to trust in various institutions, and moderate cultural identity. Table 4 displays the specific demographics.

**Table 4:** Descriptive Information of Cohort 1 Participants at Pre-Intervention

Variable	Cronbach’s alpha	n	M	SD
Age		8	37.62	17.25
NZDepi (8 items; 0–8 with 8 being highest deprivation)		6	2.83	2.48
Food Insecurity (3 items, 1–3 with 3 being lowest insecurity)	.73	7	2.48	0.50
Trust in Institutions (7 items; 0–10 with 10 as highest trust)	.91	6	4.88	2.19
Cultural identity (2 items; 1–4 with 4 being highest identity)	.69	6	2.83	0.61

#### Outcome measures

There was an 85% retention rate (35 retained) for the overall sample, and 88% for the Māori men. Some of the Māori men did not complete all of the post-intervention measures. Due to the small sample size in this cohort, there were no statistically significant differences. However, there were large effects for weight loss (Cohen’s d = 1.04; 4% reduction), BMI (d = 1.08), medium effects for self-rated health (d = .65) and HRQOL (d = .59), and small effects for nutrition (d = .21). Table 5 displays the outcomes for the 1st cohort.

**Table 5:** Pre-intervention/Post-Intervention outcomes for Cohort 1

Outcome	Cronbach’s alpha		n	Pre		Post	
	Pre	Post		M	SD	M	SD
Weight (kg) at 12 weeks			6	126.16	20.04	121.34	19.93
BMI at 12 weeks			6	39.85	5.37	38.36	5.69
Self-rated health (100-point scale with 100 as highest)			6	56.67	29.44	70.00	24.49
HRQOL (100-point scale)	.83	.87	5	63.57	15.26	72.43	20.50
Health service utilisation (6 items; 0–6 with 6 as highest utilisation)			3	2.00	2.0	2.33	3.21
Total days with 30 min moderate/15 min vigorous			2	4.00	0.00	3.50	2.21
Nutrition (9 items; 1–6 with 1 = highest nutrition)	.83	.83	4	3.44	0.45	3.17	0.90
Social support (2 items; 1–5 with 1 = highest support)	.89	.93	5	1.60	0.55	1.50	0.50
Readiness to change (3 items; 1–5 with 1 = highest commitment to change)	.69	.88	3	1.78	0.38	1.56	0.38
Efficacy to change (3 items; 1–5 with 1 = highest efficacy)	.79	.66	3	1.78	0.19	1.56	0.51

**p < .01, *p < .05

#### Post-intervention open-ended results

The open-ended results were collected through a self-report survey with seven men responding. Participants generally reported a positive impact; specifically 86% said the intervention resulted in positive health gains for themselves and all with family (*n* = 6) saying it had positive impacts on family activity and eating. The participants also reported positive feelings about the programme particularly around doing the activities with others. Table 6 presents quotes to illustrate the impact of the intervention.

**Table 6:** Cohort 1 Quotations about the Impact of the Intervention

Positive Impacts for Individuals	Positive Impacts for Whānau	Why It Worked
Involved in oranga tinana activities now [programme to improve health]	Group activities; healthy eating (most of the time)	Eating healthier and exercise
I now exercise everyday	Everyone in my household are getting into their exercise	One big whānau trying to lose weight.
Awesome and good outcomes	Whānau are pretty happy	Loved everything and the most effective was the daily exercise

### Cohort 2

#### Demographics

A total of 24 participants enrolled for the second cohort; all were male and Māori. The average age was just above 40 and the sample had relatively low food insecurity and deprivation scores. They expressed low trust in various institutions, high cultural identity, and high levels of readiness and efficacy to change. Table 7 displays the specific demographics.

**Table 7:** Descriptive Information of Cohort 2 Participants at Pre-Intervention

Variable	Cronbach’s alpha	n	M	SD
Age		24	40.58	7.67
NZDepi (8 items; 0–8 with 8 being highest deprivation)	–	8	1.88	1.36
Food Insecurity (3 items, 1–3 with 3 being lowest insecurity)	.89	24	2.50	0.67
Trust in Institutions (7 items; 0–10 with 10 as highest trust)	.93	22	3.35	2.13
Cultural identity (3 items; 1–4 with 4 being highest identity)	.69	18	3.11	0.60
Efficacy in making change (1–5 with 1 = highest efficacy)	.91	24	1.61	0.75
Readiness to make change (1–5 with 1 = highest readiness)	.90	9	1.74	0.72

#### Outcome measures

There was a 100% retention rate in the programme with all men completing initial and final weigh ins. However, only 8 men completed the post-intervention surveys even though all 24 completed the pre-intervention survey. Participation throughout the weeks was variable; there was an average of 14.71 (SD = 11.39) visits with the kaiarahi during the programme. The median of visits was 10.5 with five men (20.8%) having five or fewer visits with the kaiarahi.

There was a statistically significant weight loss in the cohort with a 4.7% average loss (d = 1.16). BMI was also significantly reduced (d = 1.15). HRQOL had more than a 30-point increase (d = 1.7). Further, self-rated health more than doubled (d = 2.0). Total days of 30 min of moderate or 15 min of vigorous activity and nutrition both improved, but did not achieve statistical significance because of the small response rates in the post-intervention survey. Table 8 displays the outcomes for the 2nd cohort.

**Table 8:** Pre-intervention/Post-Intervention outcomes for Cohort 2

Outcome	Cronbach’s alpha		N	Pre		Post	
	Pre	Post		M	SD	M	SD
Weight (kg) at 12 weeks			24	123.63	22.74	**117.79	20.06
BMI at 12 weeks			24	37.94	7.02	**36.15	6.18
Self-rated health (100-point scale with 100 as highest)			8	32.50	18.32	**80.00	15.11
HRQOL (100-point scale)	.92	.83	8	61.52	19.33	**96.07	7.01
Total days with 30 min moderate/15 min vigorous			8	3.13	3.18	4.13	1.55
Nutrition (9 items; 1–6 with 1 = highest nutrition)	.66	89	4	3.42	0.44	2.33	0.92

**p < .01, *p < .05

#### Post-intervention open-ended results

The open-ended results were collected through a self-report survey; 10 participants responded. Participants generally reported a positive impact; specifically 80% said the intervention resulted in positive health gains for themselves and their whānau with one saying no change and one unsure. The participants reported that having the kaiarahi was key to their success. They also mentioned that the health checks and information from the nurse was very important. Overall, they loved the programme and strongly disliked the paperwork (and hence the low response rate post-intervention). Table 9 presents quotes to illustrate the impact of the intervention.

**Table 9:** Cohort 2 Quotations about the Impact of the Intervention

Positive Impacts for Individuals	Positive Impacts for Whānau	Why It Worked
My breathing is a lot better. I sleep better and don’t snore as much.	They are happy I’m getting healthy. They are getting healthy too. We eat better and the kids are playing outside more. I think we are happier.	Knowing I was getting weighed and measured by the nurse. She had good information.
I feel way better and have more energy. I’m eating less rubbish. I feel fitter.	My whānau are proud that I’m making good changes and they awhi (support) me and I awhi them. They are doing good. I think we are healthier and talking more.	The nurse giving information about food and how she spoke to me.
I eat healthier kai (food). Less fat and sugar. I am feeling healthier	We eat better as a whānau. Eat more veggies and drink more water. My Mrs. loves it.	Someone there watching me and helping
Don’t get as shy like I used to.	Me and my whānau talk and get out more. The kids are playing outside heaps more now. We just talk about kai (food) now and how I want us to be eating better kai and doing more things without phones and computers.	Talking with us all the time.

## Discussion

The purpose of this study was to present a case of a partnership involving a Māori community health provider and a university research team to develop a 12-week lifestyle intervention through the HPW co-design/participatory research process. This study documented the co-design process and presented findings about two pilot study interventions which approached a clinically significant weight loss of 5% [48]. This section explores these findings in the context of the extant literature and identifies key lessons learnt that may be useful for consideration by other projects targeting health gains in Māori and other Indigenous communities.

The findings of the intervention has some consistency with previous work demonstrating the positive impacts of culturally adapting evidence-based lifestyle interventions [49, 50]. The current intervention used cultural elements and foundations such as tuakana/kaiarahi and whānau along with physical activities that are appreciated by the community to achieve its goals. Participants reflected positively about the fit of the programme to their values. The use of tuakana and kaiarahi is consistent with the literature on community health workers (CHW) [51, 52]. CHW are frequently employed with Indigenous communities in order to connect culturally with participants and systematic reviews demonstrate positive health gains from interventions delivered by CHW [31, 32].

The current findings are also supportive of the benefits of using participatory research approaches to develop and adapt lifestyle interventions for risk factors associated with diabetes, cardiovascular disease, and obesity [20–23]. Participatory research approaches such as CBPR and HPW are frequently used with Indigenous communities to address health equity and improve health [17, 18, 53, 54]. Participatory approaches establish strong relationships and emphasise community strengths that help to overcome historical mistrust and also build capacity and change systems and policies for community benefit [55–57].

### Lessons learnt

Despite challenges of not being able to implement our originally designed intervention, we had some successes as well and attribute these to the project being guided by the HPW principles. Several partners commented that HPW principles are critical to achieving health gains for Māori communities. We built strong relationships and established trust which is a key outcome in and of itself [47]. Such relationships and trust take time and there needs to be sufficient co-design/participatory process to establish the relationship. Co-design is a common term in Aotearoa for describing various forms of participatory research [58]. Beyond co-design only at the beginning, a project needs co-implementation, co-evaluation, and co-dissemination as was done in this project.

Another key lesson is the challenge of implementing innovative interventions in the field, particularly when involving multiple organisational partners. As was noted, our original intervention focussed on an integrative health care solution for a disenfranchised population. However, with two key organisations experiencing major challenges that inhibited their ability to participate in the intervention, there was a need to revise the intervention focus and thus required flexibility from the research team. In addressing health equity issues, innovative and complex programmes are needed; however, they are not without challenges. The co-design process using the HPW framework offers flexibility to adapt to these challenges although one of the compromises that may need to be made is to traditional rigour in research design. The advantages in implementation process and acceptability of the research for the community may outweigh the focus on scientific rigour.

It was difficult to recruit Māori men who were working full-time which is consistent with prior research on this population [8, 25]. While we experienced a lot of interest and desire for change, the men’s life situations made it difficult to tailor a programme that met their needs. There was a great desire for whānau-based efforts and group activities, some of which we were able to meet. The kaiarahi was a very important role that could meet individual needs of participants according to their timeframes and this type of tailored approach is our recommendation for the future.

Existing organisational and systems processes for obtaining resources were at times difficult to navigate. For example, we were not able to complete medical screens for the first cohort once the PHO was not involved. Our lesson here is that we probably should have done more integrated knowledge translation (IKT) earlier in the project. While we included the DHB in the early stages, we focussed on the PHO-level throughout the process. Structurally, the DHB has more resources and influence that could have been better leveraged.

Additionally, a key concern of community members is whether the programmes will be sustained for the long-term. Many participants wondered if the programme worked whether they could count on it being around next year. We were honest about the timeline of the research and made efforts to find continued support. We have commitment by Poutiri and a local aquatic centre to continue support for the intervention beyond this period. Poutiri’s Board of Trustees recently committed funding to continue the intervention from the 2nd cohort. The PHO is also interested in supporting the project now that they have stabilised. We remain in discussions to see if we can sustain these efforts. These discussions are a result of the co-design and IKT efforts following HPW principles.

### Limitations

The study has a few key limitations. First, the two cohorts were single group designs without a comparison group. Second, poor response rates to the questionnaire in the second cohort limit the power for the pre−/post-intervention comparisons. Third, the lack of clinical measures (e.g., HbA1c) limit conclusions about the overall effectiveness of the interventions despite significant reduction in weight.

## Conclusions

Overall, we deemed this pilot study to develop/adapt a lifestyle intervention to reduce risk factors for diabetes and related conditions for Māori men a success. We conclude that a customised and reflexive approach contributed to retention and some positive outcomes. An effective intervention will need to be tailored and adapted to fit the needs of the participants and communities. A willingness to co-create and adapt is key to building rapport and trust with providers and communities. It also creates a commitment to sustain the work.

Further, the HPW framework is useful for guiding this co-design work, particularly as a self-monitoring tool during design and implementation. Research partners can use the framework and related evaluation tool to conduct process evaluation and ensure that principles are being followed. The framework should be viewed as fluid as it can be influenced by changes in people, communities and even processes. If reviewed at regular intervals the framework can be used as a tool for continuous improvement in implementation. The framework also supported accountability of self, giving participants the opportunity to grow their knowledge of nutrition, exercise, and highlighting their own accountability in maintaining their achievements from the programme.

Finally, long-term structural changes (e.g., improving health equity) take a multi-pronged, systems focus. Such an approach is complex and has many challenges that make these high risk-high reward approaches. However, positive gains and relationships help to sustain these efforts and have great potential for long-term success.

## Data Availability

The datasets used and/or analysed during the current study are available from the corresponding author on reasonable request.

## Abbreviations

BMI: Body mass index
CBPR: Community-based participatory research
CHW: Community health worker
HPW: He Pikinga Waiora Implementation Framework
HRQOL: Health-related quality of life

## Māori Words

Aotearoa: New Zealand
He Pikinga Waiora: Enhancing wellbeing
Kaiarahi: Guide; community health worker
Kaupapa Māori: Research by Māori for Māori
Māori: Indigenous people of New Zealand
Pākehā: New Zealand European
Te Tiriti or Waitangi: Treaty of Waitangi
Tuakana: Senior mentor
Whānau: Extended family
